# Crystal structure of diethyl 2-amino-6-[(thio­phen-3-yl)ethyn­yl]azulene-1,3-di­carboxyl­ate

**DOI:** 10.1107/S2056989015003898

**Published:** 2015-02-28

**Authors:** Sebastian Förster, Wilhelm Seichter, Edwin Weber

**Affiliations:** a Institut für Organische Chemie, TU Bergakademie Freiberg, Leipziger Strasse 29, D-09596 Freiberg/Sachsen, Germany

**Keywords:** crystal structure, azulene, thio­phene, 3-thienylethyn­yl, hydrogen bonds, C—H⋯π inter­actions

## Abstract

The title compound, C_22_H_19_NO_4_S, has an almost planar geometry supported by intra­molecular N—H⋯O and C—H⋯O hydrogen bonds. The thio­phene ring is inclined to the azulene ring by 4.85 (16)°, while the eth­oxy­carbonyl groups are inclined to the azulene ring by 7.0 (2) and 5.7 (2)°. In the crystal, mol­ecules are linked by pairs of N—H⋯O hydrogen bonds, forming inversion dimers with an *R*_2_^2^(12) ring motif. The dimers are linked *via* C—H⋯π inter­actions, forming sheets parallel to (10-1).

## Related literature   

For the synthesis of the title compound concerning the azulene-derived starting material, see: McDonald *et al.* (1976 ▸). For the background of this work and for the synthesis of related compounds, see: Xia *et al.* (2014 ▸); Förster *et al.* (2012 ▸). For related structures, see: Förster *et al.* (2014 ▸); Shoji *et al.* (2013 ▸). For C—H⋯π contacts, see: Nishio *et al.* (2009 ▸).
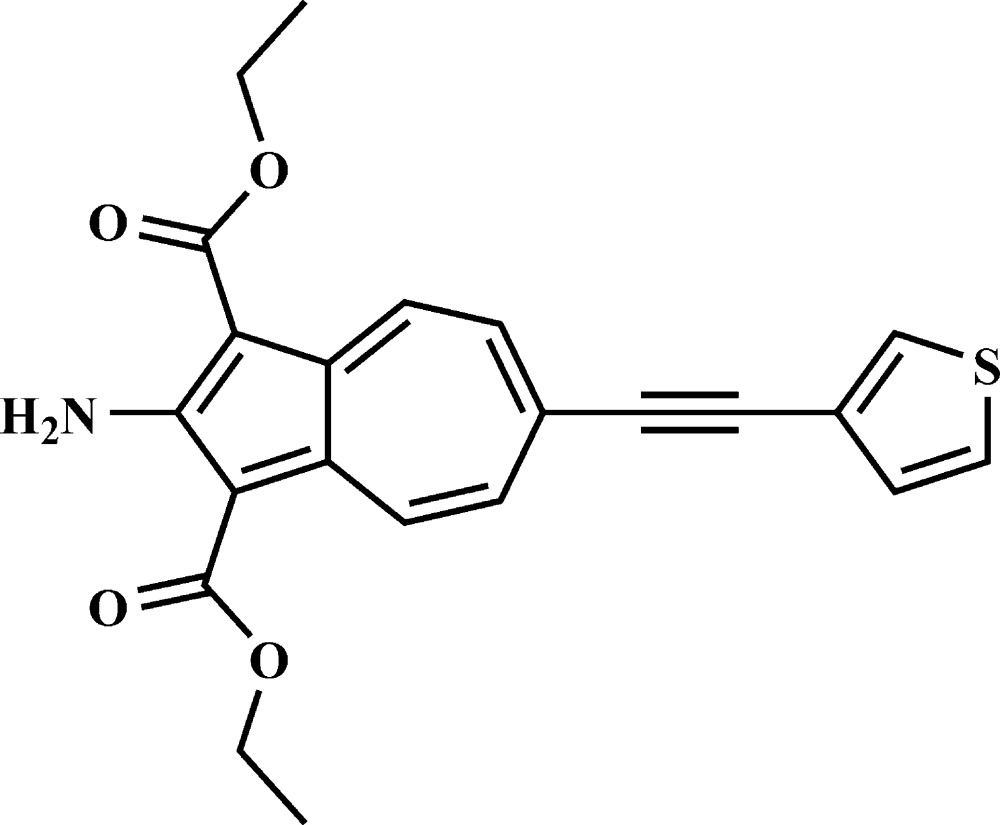


## Experimental   

### Crystal data  

C_22_H_19_NO_4_S
*M* *_r_* = 393.44
Monoclinic, 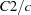

*a* = 22.2429 (5) Å
*b* = 5.5039 (1) Å
*c* = 32.8340 (8) Å
β = 102.914 (1)°
*V* = 3917.96 (15) Å^3^
*Z* = 8
Mo *K*α radiation
μ = 0.19 mm^−1^
*T* = 296 K
0.50 × 0.14 × 0.04 mm

### Data collection  

Bruker SMART CCD area-detector diffractometer
Absorption correction: multi-scan (*SADABS*; Bruker, 2007 ▸) *T*_min_ = 0.909, *T*_max_ = 0.992
18444 measured reflections
3679 independent reflections
2441 reflections with *I* > 2σ(*I*)
*R*_int_ = 0.032

### Refinement  

*R*[*F* ^2^ > 2σ(*F* ^2^)] = 0.067
*wR*(*F* ^2^) = 0.242
*S* = 1.04
3679 reflections
255 parameters
H-atom parameters constrained
Δρ_max_ = 0.68 e Å^−3^
Δρ_min_ = −0.69 e Å^−3^

### 

Data collection: *APEX2* (Bruker, 2007 ▸); cell refinement: *SAINT* (Bruker, 2007 ▸); data reduction: *SAINT*; program(s) used to solve structure: *SHELXS97* (Sheldrick, 2008 ▸); program(s) used to refine structure: *SHELXL2013* (Sheldrick, 2015 ▸); molecular graphics: *ORTEP-3 for Windows* (Farrugia, 2012 ▸); software used to prepare material for publication: *SHELXTL* (Sheldrick, 2008 ▸).

## Supplementary Material

Crystal structure: contains datablock(s) I, Global. DOI: 10.1107/S2056989015003898/su5088sup1.cif

Structure factors: contains datablock(s) I. DOI: 10.1107/S2056989015003898/su5088Isup2.hkl

Supporting information file. DOI: 10.1107/S2056989015003898/su5088Isup3.cml

**Fig. 1.**
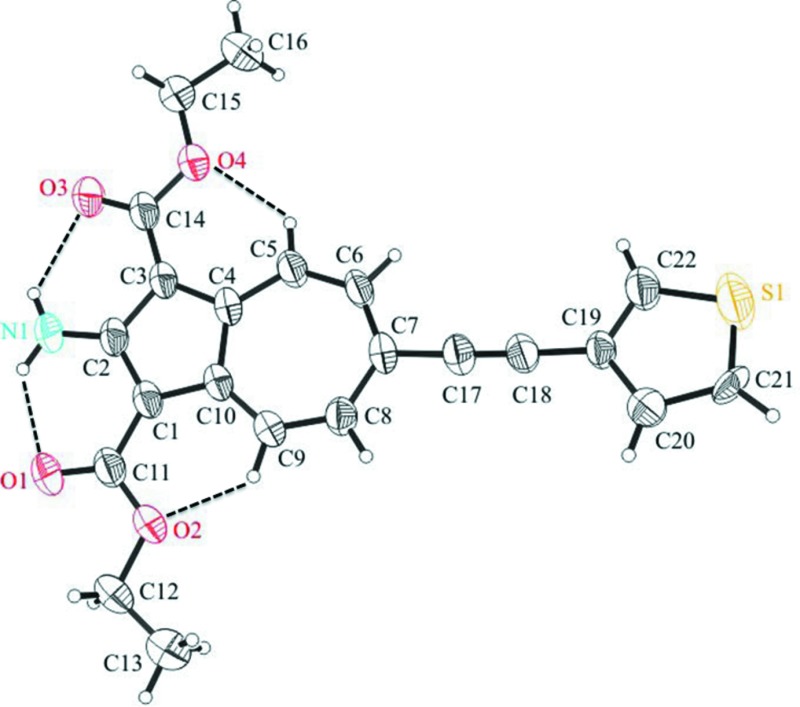
A view of the molecular structure of the title compound, with atom labelling. Displacement ellipsoids are drawn at the 50% probability level. The hydrogen bonds are shown as dashed lines (see Table 1 for details).

**Fig. 2.**
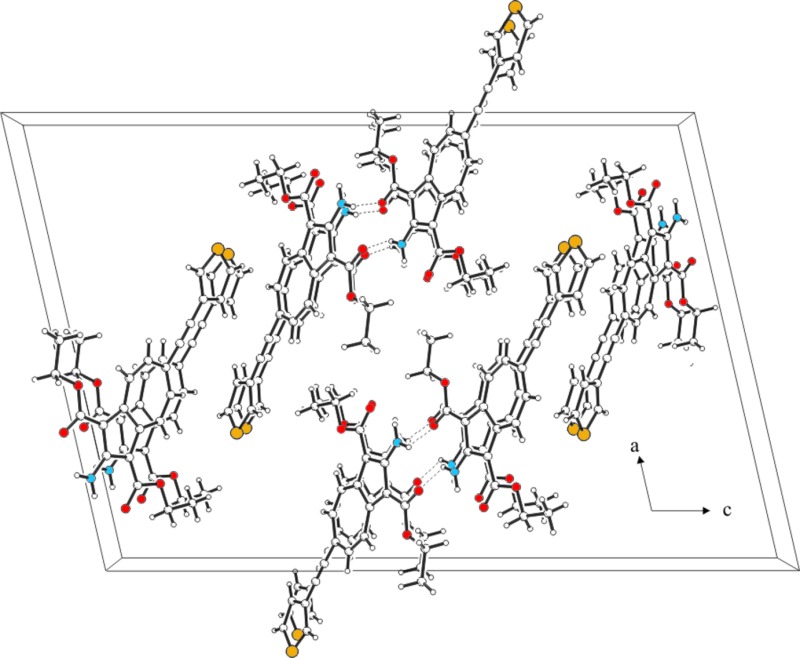
Crystal packing of the title compound viewed along the b-axis. Hydrogen bonds are shown as dashed lines (see Table 1 for details).

. DOI: 10.1107/S2056989015003898/su5088fig1.tif
A view of the mol­ecular structure of the title compound, with atom labelling. Displacement ellipsoids are drawn at the 50% probability level. The hydrogen bonds are shown as dashed lines (see Table 1 for details).

b . DOI: 10.1107/S2056989015003898/su5088fig2.tif
Crystal packing of the title compound viewed along the *b*-axis. Hydrogen bonds are shown as dashed lines (see Table 1 for details).

CCDC reference: 1051132

Additional supporting information:  crystallographic information; 3D view; checkCIF report

## Figures and Tables

**Table 1:** Hydrogen-bond geometry (, ) *Cg*1 is the centroid of the thiophene ring S1/C19C22.

*D*H*A*	*D*H	H*A*	*D* *A*	*D*H*A*
N1H1*B*O3	0.86	2.16	2.765(4)	127
N1H1*A*O1	0.86	2.15	2.757(4)	127
C5H5O4	0.93	2.22	2.878(4)	127
C9H9O2	0.93	2.23	2.897(4)	128
N1H1*B*O3^i^	0.86	2.21	2.959(4)	146
C8H8*Cg*1^ii^	0.93	2.92	3.714(4)	144

Symmetry codes: (i) 
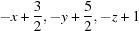
; (ii) 
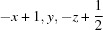
.

## supplementary crystallographic information

### S1. Synthesis and crystallization

The synthesis of the title compound was done starting from the literature known azulene derivative di­ethyl 2-amino-6-bromo­azulene-1,3-di­carboxyl­ate (McDonald *et al.*, 1976). This compound (1.0 g, 2.73 mmol) together with 3-ethynyl­thio­phene (0.33 g, 3.05 mmol) was dissolved in a mixture of 25 ml diiso­propyl­amine and 25 ml tetra­hydro­furan. After degassing of the solution, bis­(tri­phenyl­phosphane)palladium(II) chloride (32 mg, 0.045 mmol), copper(I) iodide (17 mg, 0.09 mmol) and tri­phenyl­phosphane (39 mg, 0.15 mmol) were added. The mixture was stirred for 10 h under reflux and an argon atmosphere. After evaporation of the solvent, the residue was purified by column chromatography on SiO_2_ [60 F_254_ Merck, eluent: hexane/ethyl acetate (100:1)] to yield 0.81 g (75%) of the title compound as a red solid. Analytical data: mp = 148-149°C; ^1^H-NMR: (CDCl_3_) δ/ppm = 9.02 (d, 2H, ArH, ^3^J_HH_ = 11.5 Hz), 7.86 (s, 2H, NH_2_), 7.73 (d, 2H, ArH, ^3^J_HH_ = 11.5 Hz), 7.59 (dd, 1H, ArH, ^4^J_HH_ = 2.9 Hz, ^5^J_HH_ = 1.1 Hz), 7.34 (dd, 1H, ArH, ^3^J_HH_ = 5.0 Hz, ^4^J_HH_ = 3.0 Hz), 7.23 (dd, 1H, ArH, ^3^J_HH_ = 5.0 Hz, ^5^J_HH_ = 1.1 Hz), 4.47 (q, 4H, CH_2_, ^3^J_HH_ = 7.2 Hz), 1.48 (t, 6H, CH_3_, ^3^J_HH_ = 7.2 Hz); ^13^C-NMR: (CDCl_3_) δ/ppm = 166.40 (CO), 162.53 (ArC), 145.55 (ArC), 135.24 (ArC), 129.83 (ArC), 129.82 (ArC), 129.40 (ArC), 127.81 (ArC), 125.65 (ArC), 121.92 (ArC), 100.62 (C≡C), 92.47 (C≡C), 59.98 (CH_2_), 14.63 (CH_3_); GC/MS calc.: 393.5; found: 393 [M]^+^.;EA calc.: C: 67.16 %, H: 4.87 %, N: 3.56 %, S: 8.15 %; found C: 67.10 %, H: 4.75 %, N: 3.51 %, S: 7.97 %; Crystallization by slow solvent evaporation from di­chloro­methane solution yielded suitable crystals.

### S2. Refinement

Crystal data, data collection and structure refinement details are summarized in Table 2. The C- and N-bound H atoms were fixed geometrically and treated as riding: N—H = 0.86 Å, C—H = 0.93 - 0.97 Å, with U_iso_(H) =1.5U_eq_(C) for methyl H atoms and = 1.2U_eq_(N,C) for other H atoms.

### S3. Comment

Due to the special physical properties including redox behavior, azulene is an interesting structure building block for electronic materials design (Förster *et al.*, 2012; Shoji *et al.*, 2013; Xia *et al.*, 2014). In this regard, the present tetrasubstituted azulene derivative, as the title compound, represents a promising intermediate.

The title compound has an almost planar overall geometry with maximum deviations of 0.166 (1) Å for C20 and 0.267 (4) Å for C6 (Fig. 1). In contrast to previously published related compounds (Förster *et al.*, 2014), here the amine group gives rise to the formation of two intramolecular N—H···O hydrogen bonds to the neighbouring carbonyl O atoms O1 and O3 (Table 1 and Fig. 1). Furthermore, atoms O2 and O4 establish two weaker intramolecular C—H···O hydrogen bonds to azulene hydrogen atoms (Table 1 and Fig. 1).

In the crystal, an *R*_2_^2^(12) hydrogen bonded inversion dimer motif is formed (Table 1 and Fig. 2). Along the crystallographic *b*-axis, the corresponding dimers are arranged in stacks (Fig. 2). Despite a plane to plane distance of 3.15 Å between the azulene units of consecutive molecules, no arene···arene interactions can be observed due to the lateral displacement. In direction of the crystallographic *a*- and *c*-axes, these stacks are connected *via* C—H···π contacts [Table 1; Nishio *et al.*, 2009] and weak van der Waals forces, forming a sheets parallel to (10\1).. The absence of arene···arene interactions is a rather rare phenomenon for this class of azulenes, and is probably due to packing effects (Förster *et al.*, 2014).

### Crystal data

**Table:**

C_22_H_19_NO_4_S	*F*(000) = 1648
*M**_r_* = 393.44	*D*_x_ = 1.334 Mg m^−^^3^
Monoclinic, *C*2/*c*	Mo *K*α radiation, λ = 0.71073 Å
*a* = 22.2429 (5) Å	Cell parameters from 4036 reflections
*b* = 5.5039 (1) Å	θ = 2.5–23.9°
*c* = 32.8340 (8) Å	µ = 0.19 mm^−^^1^
β = 102.914 (1)°	*T* = 296 K
*V* = 3917.96 (15) Å^3^	Rod, red
*Z* = 8	0.50 × 0.14 × 0.04 mm

### Data collection

**Table:**

Bruker SMART CCD area-detector diffractometer	3679 independent reflections
Radiation source: fine-focus sealed tube	2441 reflections with *I* > 2σ(*I*)
Plane graphite monochromator	*R*_int_ = 0.032
phi and ω scans	θ_max_ = 25.6°, θ_min_ = 2.6°
Absorption correction: multi-scan (*SADABS*; Bruker, 2007)	*h* = −24→26
*T*_min_ = 0.909, *T*_max_ = 0.992	*k* = −6→6
18444 measured reflections	*l* = −39→39

### Refinement

**Table:**

Refinement on *F*^2^	0 restraints
Least-squares matrix: full	Hydrogen site location: inferred from neighbouring sites
*R*[*F*^2^ > 2σ(*F*^2^)] = 0.067	H-atom parameters constrained
*wR*(*F*^2^) = 0.242	*w* = 1/[σ^2^(*F*_o_^2^) + (0.1341*P*)^2^ + 7.3721*P*] where *P* = (*F*_o_^2^ + 2*F*_c_^2^)/3
*S* = 1.04	(Δ/σ)_max_ < 0.001
3679 reflections	Δρ_max_ = 0.68 e Å^−^^3^
255 parameters	Δρ_min_ = −0.69 e Å^−^^3^

### Special details

**Table:**

Geometry. All e.s.d.'s (except the e.s.d. in the dihedral angle between two l.s. planes) are estimated using the full covariance matrix. The cell e.s.d.'s are taken into account individually in the estimation of e.s.d.'s in distances, angles and torsion angles; correlations between e.s.d.'s in cell parameters are only used when they are defined by crystal symmetry. An approximate (isotropic) treatment of cell e.s.d.'s is used for estimating e.s.d.'s involving l.s. planes.

### Fractional atomic coordinates and isotropic or equivalent isotropic displacement parameters (Å^2^)

**Table:**

	*x*	*y*	*z*	*U*_iso_*/*U*_eq_	
S1	0.29785 (6)	−0.1167 (3)	0.23302 (5)	0.0900 (5)	
O1	0.85377 (12)	0.5584 (6)	0.43542 (9)	0.0725 (9)	
O2	0.80576 (11)	0.3079 (6)	0.38532 (9)	0.0636 (8)	
O3	0.68603 (12)	1.1767 (5)	0.47849 (9)	0.0631 (8)	
O4	0.59102 (11)	1.0579 (5)	0.44835 (8)	0.0583 (7)	
N1	0.78370 (13)	0.9042 (6)	0.46415 (9)	0.0556 (8)	
H1A	0.8213	0.8599	0.4661	0.067*	
H1B	0.7755	1.0254	0.4785	0.067*	
C1	0.74517 (15)	0.5833 (6)	0.41350 (10)	0.0436 (8)	
C2	0.73847 (15)	0.7861 (7)	0.43904 (10)	0.0440 (8)	
C3	0.67421 (15)	0.8431 (6)	0.43263 (10)	0.0423 (8)	
C4	0.64141 (14)	0.6787 (6)	0.40310 (10)	0.0406 (8)	
C5	0.57758 (15)	0.6849 (7)	0.38555 (11)	0.0485 (9)	
H5	0.5566	0.8141	0.3943	0.058*	
C6	0.54133 (15)	0.5301 (7)	0.35757 (11)	0.0496 (9)	
H6	0.5001	0.5754	0.3494	0.060*	
C7	0.55645 (15)	0.3158 (6)	0.33947 (10)	0.0445 (8)	
C8	0.61599 (16)	0.2185 (6)	0.34362 (11)	0.0461 (8)	
H8	0.6178	0.0737	0.3294	0.055*	
C9	0.67212 (15)	0.3054 (6)	0.36566 (10)	0.0432 (8)	
H9	0.7061	0.2140	0.3628	0.052*	
C10	0.68606 (14)	0.5089 (6)	0.39161 (10)	0.0399 (8)	
C11	0.80595 (16)	0.4849 (7)	0.41321 (11)	0.0522 (9)	
C12	0.86442 (18)	0.1954 (10)	0.38488 (16)	0.0778 (14)	
H12A	0.8817	0.1216	0.4118	0.093*	
H12B	0.8933	0.3163	0.3792	0.093*	
C13	0.8535 (2)	0.0097 (10)	0.35201 (16)	0.0811 (14)	
H13A	0.8238	−0.1055	0.3573	0.122*	
H13B	0.8915	−0.0725	0.3518	0.122*	
H13C	0.8381	0.0856	0.3254	0.122*	
C14	0.65276 (15)	1.0391 (7)	0.45498 (11)	0.0465 (8)	
C15	0.56708 (18)	1.2514 (8)	0.46980 (13)	0.0619 (11)	
H15A	0.5837	1.4066	0.4636	0.074*	
H15B	0.5781	1.2254	0.4998	0.074*	
C16	0.4981 (2)	1.2477 (11)	0.45432 (17)	0.0881 (16)	
H16A	0.4879	1.2725	0.4246	0.132*	
H16B	0.4801	1.3748	0.4677	0.132*	
H16C	0.4823	1.0935	0.4607	0.132*	
C17	0.50538 (16)	0.1840 (7)	0.31426 (11)	0.0500 (9)	
C18	0.46201 (16)	0.0786 (7)	0.29443 (11)	0.0497 (9)	
C19	0.40854 (15)	−0.0390 (6)	0.27000 (11)	0.0430 (8)	
C20	0.40753 (18)	−0.2579 (7)	0.24830 (13)	0.0596 (10)	
H20	0.4423	−0.3521	0.2484	0.072*	
C21	0.34319 (18)	−0.3231 (6)	0.22455 (11)	0.0483 (9)	
H21	0.3322	−0.4599	0.2079	0.058*	
C22	0.35034 (18)	0.0568 (8)	0.26439 (14)	0.0636 (11)	
H22	0.3412	0.2007	0.2765	0.076*	

### Atomic displacement parameters (Å^2^)

**Table:**

	*U*^11^	*U*^22^	*U*^33^	*U*^12^	*U*^13^	*U*^23^
S1	0.0527 (7)	0.0981 (10)	0.1083 (11)	−0.0162 (6)	−0.0053 (7)	−0.0110 (8)
O1	0.0303 (14)	0.104 (2)	0.0759 (19)	−0.0053 (14)	−0.0025 (13)	−0.0228 (17)
O2	0.0318 (13)	0.084 (2)	0.0702 (17)	0.0043 (13)	0.0009 (12)	−0.0169 (15)
O3	0.0433 (15)	0.0706 (18)	0.0707 (17)	−0.0129 (13)	0.0025 (12)	−0.0237 (14)
O4	0.0364 (14)	0.0734 (17)	0.0620 (16)	−0.0051 (12)	0.0043 (11)	−0.0207 (14)
N1	0.0323 (15)	0.073 (2)	0.0567 (18)	−0.0109 (15)	−0.0012 (13)	−0.0119 (16)
C1	0.0308 (17)	0.055 (2)	0.0426 (17)	−0.0058 (15)	0.0028 (13)	0.0032 (16)
C2	0.0357 (17)	0.056 (2)	0.0380 (16)	−0.0117 (15)	0.0027 (13)	0.0022 (15)
C3	0.0331 (17)	0.0487 (18)	0.0414 (17)	−0.0068 (14)	0.0006 (13)	0.0009 (15)
C4	0.0324 (17)	0.0441 (17)	0.0406 (17)	−0.0061 (14)	−0.0013 (13)	0.0040 (14)
C5	0.0342 (18)	0.049 (2)	0.057 (2)	−0.0005 (15)	−0.0005 (15)	−0.0043 (17)
C6	0.0277 (17)	0.056 (2)	0.059 (2)	−0.0017 (15)	−0.0034 (15)	−0.0045 (17)
C7	0.0349 (18)	0.0494 (19)	0.0436 (17)	−0.0083 (15)	−0.0032 (14)	0.0017 (15)
C8	0.0411 (19)	0.0468 (19)	0.0472 (18)	−0.0050 (15)	0.0032 (15)	−0.0038 (15)
C9	0.0321 (17)	0.0488 (18)	0.0463 (18)	0.0002 (14)	0.0036 (14)	0.0026 (15)
C10	0.0313 (17)	0.0461 (18)	0.0398 (16)	−0.0043 (14)	0.0026 (13)	0.0067 (14)
C11	0.0364 (19)	0.069 (2)	0.0487 (19)	−0.0035 (18)	0.0037 (15)	0.0011 (19)
C12	0.035 (2)	0.106 (4)	0.088 (3)	0.013 (2)	0.006 (2)	−0.022 (3)
C13	0.057 (3)	0.095 (4)	0.090 (3)	0.011 (2)	0.014 (2)	−0.017 (3)
C14	0.0368 (18)	0.055 (2)	0.0439 (18)	−0.0084 (16)	0.0010 (14)	0.0020 (16)
C15	0.048 (2)	0.077 (3)	0.059 (2)	0.000 (2)	0.0099 (18)	−0.014 (2)
C16	0.048 (3)	0.120 (4)	0.094 (4)	0.013 (3)	0.012 (2)	−0.023 (3)
C17	0.039 (2)	0.053 (2)	0.054 (2)	−0.0055 (16)	0.0012 (16)	−0.0018 (17)
C18	0.0378 (19)	0.052 (2)	0.055 (2)	−0.0006 (16)	0.0017 (16)	−0.0043 (17)
C19	0.0345 (17)	0.0400 (17)	0.0511 (19)	−0.0072 (14)	0.0027 (14)	−0.0063 (15)
C20	0.050 (2)	0.054 (2)	0.074 (3)	−0.0010 (18)	0.0123 (19)	−0.015 (2)
C21	0.069 (2)	0.0315 (16)	0.0493 (19)	−0.0200 (16)	0.0234 (17)	−0.0221 (15)
C22	0.045 (2)	0.055 (2)	0.085 (3)	−0.0013 (18)	0.000 (2)	−0.018 (2)

### Geometric parameters (Å, º)

**Table:**

S1—C21	1.584 (4)	C8—C9	1.381 (5)
S1—C22	1.673 (4)	C8—H8	0.9300
O1—C11	1.216 (4)	C9—C10	1.400 (5)
O2—C11	1.336 (5)	C9—H9	0.9300
O2—C12	1.447 (5)	C12—C13	1.467 (7)
O3—C14	1.209 (4)	C12—H12A	0.9700
O4—C14	1.345 (4)	C12—H12B	0.9700
O4—C15	1.443 (5)	C13—H13A	0.9600
N1—C2	1.320 (4)	C13—H13B	0.9600
N1—H1A	0.8600	C13—H13C	0.9600
N1—H1B	0.8600	C15—C16	1.504 (6)
C1—C10	1.411 (4)	C15—H15A	0.9700
C1—C2	1.424 (5)	C15—H15B	0.9700
C1—C11	1.459 (5)	C16—H16A	0.9600
C2—C3	1.432 (5)	C16—H16B	0.9600
C3—C4	1.405 (5)	C16—H16C	0.9600
C3—C14	1.445 (5)	C17—C18	1.188 (5)
C4—C5	1.408 (5)	C18—C19	1.432 (5)
C4—C10	1.473 (5)	C19—C22	1.371 (5)
C5—C6	1.374 (5)	C19—C20	1.397 (5)
C5—H5	0.9300	C20—C21	1.512 (5)
C6—C7	1.395 (5)	C20—H20	0.9300
C6—H6	0.9300	C21—H21	0.9300
C7—C8	1.407 (5)	C22—H22	0.9300
C7—C17	1.443 (5)		
			
C21—S1—C22	97.7 (2)	O2—C12—H12A	110.2
C11—O2—C12	117.0 (3)	C13—C12—H12A	110.2
C14—O4—C15	116.9 (3)	O2—C12—H12B	110.2
C2—N1—H1A	120.0	C13—C12—H12B	110.2
C2—N1—H1B	120.0	H12A—C12—H12B	108.5
H1A—N1—H1B	120.0	C12—C13—H13A	109.5
C10—C1—C2	108.6 (3)	C12—C13—H13B	109.5
C10—C1—C11	130.4 (3)	H13A—C13—H13B	109.5
C2—C1—C11	120.9 (3)	C12—C13—H13C	109.5
N1—C2—C1	126.0 (3)	H13A—C13—H13C	109.5
N1—C2—C3	125.5 (3)	H13B—C13—H13C	109.5
C1—C2—C3	108.5 (3)	O3—C14—O4	120.8 (3)
C4—C3—C2	107.9 (3)	O3—C14—C3	124.6 (3)
C4—C3—C14	130.7 (3)	O4—C14—C3	114.6 (3)
C2—C3—C14	121.4 (3)	O4—C15—C16	106.6 (3)
C3—C4—C5	126.0 (3)	O4—C15—H15A	110.4
C3—C4—C10	108.0 (3)	C16—C15—H15A	110.4
C5—C4—C10	125.9 (3)	O4—C15—H15B	110.4
C6—C5—C4	130.2 (3)	C16—C15—H15B	110.4
C6—C5—H5	114.9	H15A—C15—H15B	108.6
C4—C5—H5	114.9	C15—C16—H16A	109.5
C5—C6—C7	130.4 (3)	C15—C16—H16B	109.5
C5—C6—H6	114.8	H16A—C16—H16B	109.5
C7—C6—H6	114.8	C15—C16—H16C	109.5
C6—C7—C8	126.3 (3)	H16A—C16—H16C	109.5
C6—C7—C17	115.8 (3)	H16B—C16—H16C	109.5
C8—C7—C17	117.9 (3)	C18—C17—C7	177.6 (4)
C9—C8—C7	129.8 (3)	C17—C18—C19	177.6 (4)
C9—C8—H8	115.1	C22—C19—C20	110.8 (3)
C7—C8—H8	115.1	C22—C19—C18	122.8 (3)
C8—C9—C10	130.4 (3)	C20—C19—C18	126.3 (3)
C8—C9—H9	114.8	C19—C20—C21	111.9 (3)
C10—C9—H9	114.8	C19—C20—H20	124.0
C9—C10—C1	126.6 (3)	C21—C20—H20	124.0
C9—C10—C4	126.4 (3)	C20—C21—S1	107.8 (2)
C1—C10—C4	106.9 (3)	C20—C21—H21	126.1
O1—C11—O2	121.5 (3)	S1—C21—H21	126.1
O1—C11—C1	124.1 (4)	C19—C22—S1	111.8 (3)
O2—C11—C1	114.5 (3)	C19—C22—H22	124.1
O2—C12—C13	107.7 (3)	S1—C22—H22	124.1
			
C10—C1—C2—N1	179.6 (3)	C3—C4—C10—C9	174.5 (3)
C11—C1—C2—N1	0.1 (5)	C5—C4—C10—C9	−9.2 (5)
C10—C1—C2—C3	−1.0 (4)	C3—C4—C10—C1	−2.4 (4)
C11—C1—C2—C3	179.5 (3)	C5—C4—C10—C1	173.9 (3)
N1—C2—C3—C4	178.8 (3)	C12—O2—C11—O1	4.5 (6)
C1—C2—C3—C4	−0.6 (4)	C12—O2—C11—C1	−177.3 (4)
N1—C2—C3—C14	−1.7 (5)	C10—C1—C11—O1	−175.9 (4)
C1—C2—C3—C14	178.9 (3)	C2—C1—C11—O1	3.4 (6)
C2—C3—C4—C5	−174.4 (3)	C10—C1—C11—O2	5.9 (6)
C14—C3—C4—C5	6.2 (6)	C2—C1—C11—O2	−174.7 (3)
C2—C3—C4—C10	1.8 (4)	C11—O2—C12—C13	−179.6 (4)
C14—C3—C4—C10	−177.6 (3)	C15—O4—C14—O3	1.3 (5)
C3—C4—C5—C6	−178.0 (4)	C15—O4—C14—C3	−179.8 (3)
C10—C4—C5—C6	6.4 (6)	C4—C3—C14—O3	−176.4 (4)
C4—C5—C6—C7	2.8 (7)	C2—C3—C14—O3	4.2 (6)
C5—C6—C7—C8	−5.8 (7)	C4—C3—C14—O4	4.7 (5)
C5—C6—C7—C17	174.0 (4)	C2—C3—C14—O4	−174.7 (3)
C6—C7—C8—C9	0.1 (6)	C14—O4—C15—C16	174.9 (4)
C17—C7—C8—C9	−179.7 (4)	C22—C19—C20—C21	0.9 (5)
C7—C8—C9—C10	3.0 (6)	C18—C19—C20—C21	−177.9 (3)
C8—C9—C10—C1	179.2 (4)	C19—C20—C21—S1	−0.5 (4)
C8—C9—C10—C4	2.8 (6)	C22—S1—C21—C20	0.0 (3)
C2—C1—C10—C9	−174.9 (3)	C20—C19—C22—S1	−0.9 (5)
C11—C1—C10—C9	4.6 (6)	C18—C19—C22—S1	177.9 (3)
C2—C1—C10—C4	2.0 (4)	C21—S1—C22—C19	0.5 (4)
C11—C1—C10—C4	−178.5 (3)		

### Hydrogen-bond geometry (Å, º)

Cg1 is the centroid of the thiophene ring S1/C19–C22.

**Table:**

*D*—H···*A*	*D*—H	H···*A*	*D*···*A*	*D*—H···*A*
N1—H1*B*···O3	0.86	2.16	2.765 (4)	127
N1—H1*A*···O1	0.86	2.15	2.757 (4)	127
C5—H5···O4	0.93	2.22	2.878 (4)	127
C9—H9···O2	0.93	2.23	2.897 (4)	128
N1—H1*B*···O3^i^	0.86	2.21	2.959 (4)	146
C8—H8···*Cg*1^ii^	0.93	2.92	3.714 (4)	144

Symmetry codes: (i) −*x*+3/2, −*y*+5/2, −*z*+1; (ii) −*x*+1, *y*, −*z*+1/2.
