# Supplementary figures and images for: Crystal structure of diethyl 2-amino-6-[(thio­phen-3-yl)ethyn­yl]azulene-1,3-di­carboxyl­ate
Source: Acta Crystallogr E Crystallogr Commun. 2015 Feb 28;71(Pt 3):o212–3. doi: 10.1107/S2056989015003898 (PMC4350707; doi:10.1107/S2056989015003898)

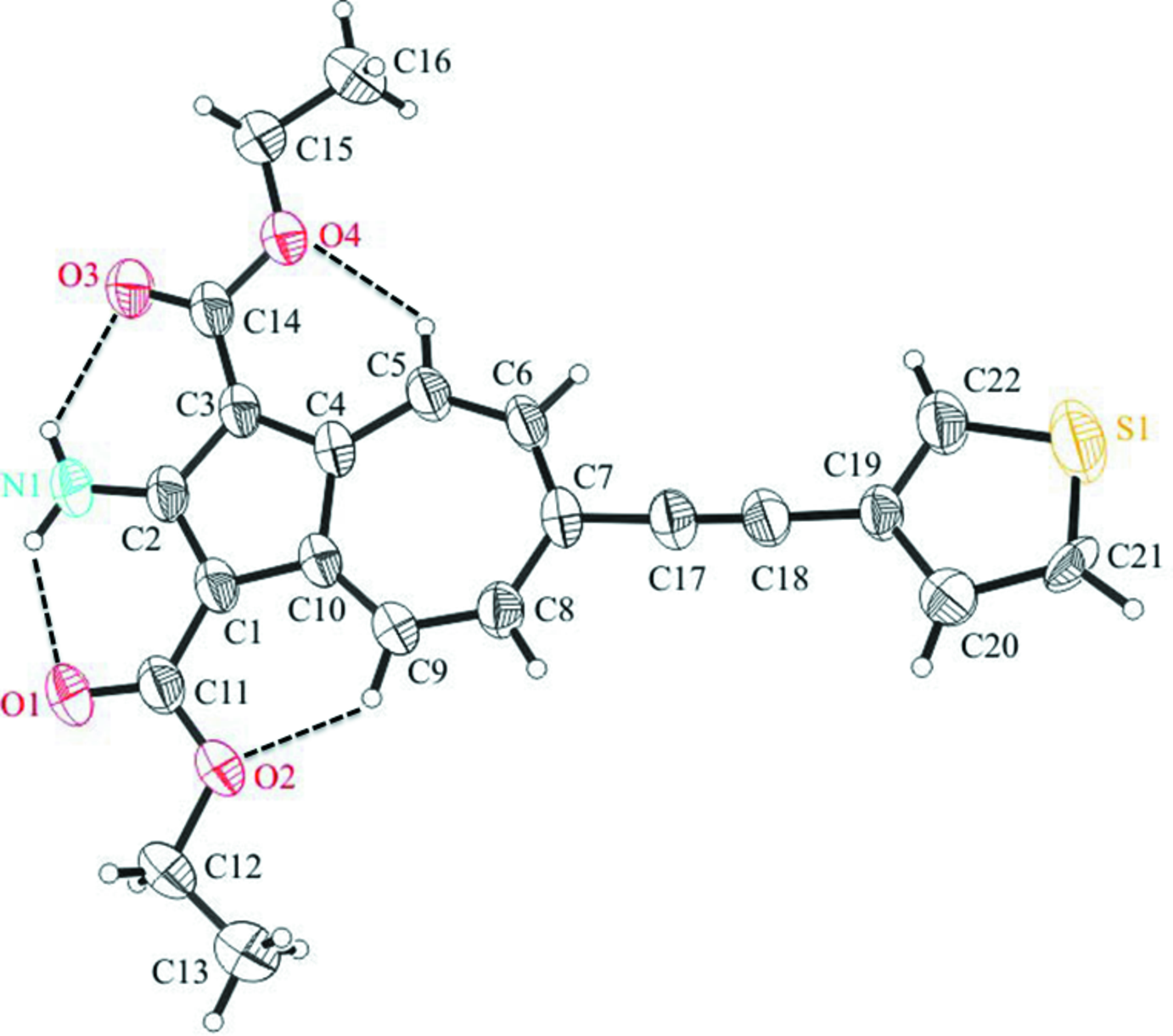

Supplement: Supplementary file 4 [file e-71-0o212-fig1.tif]

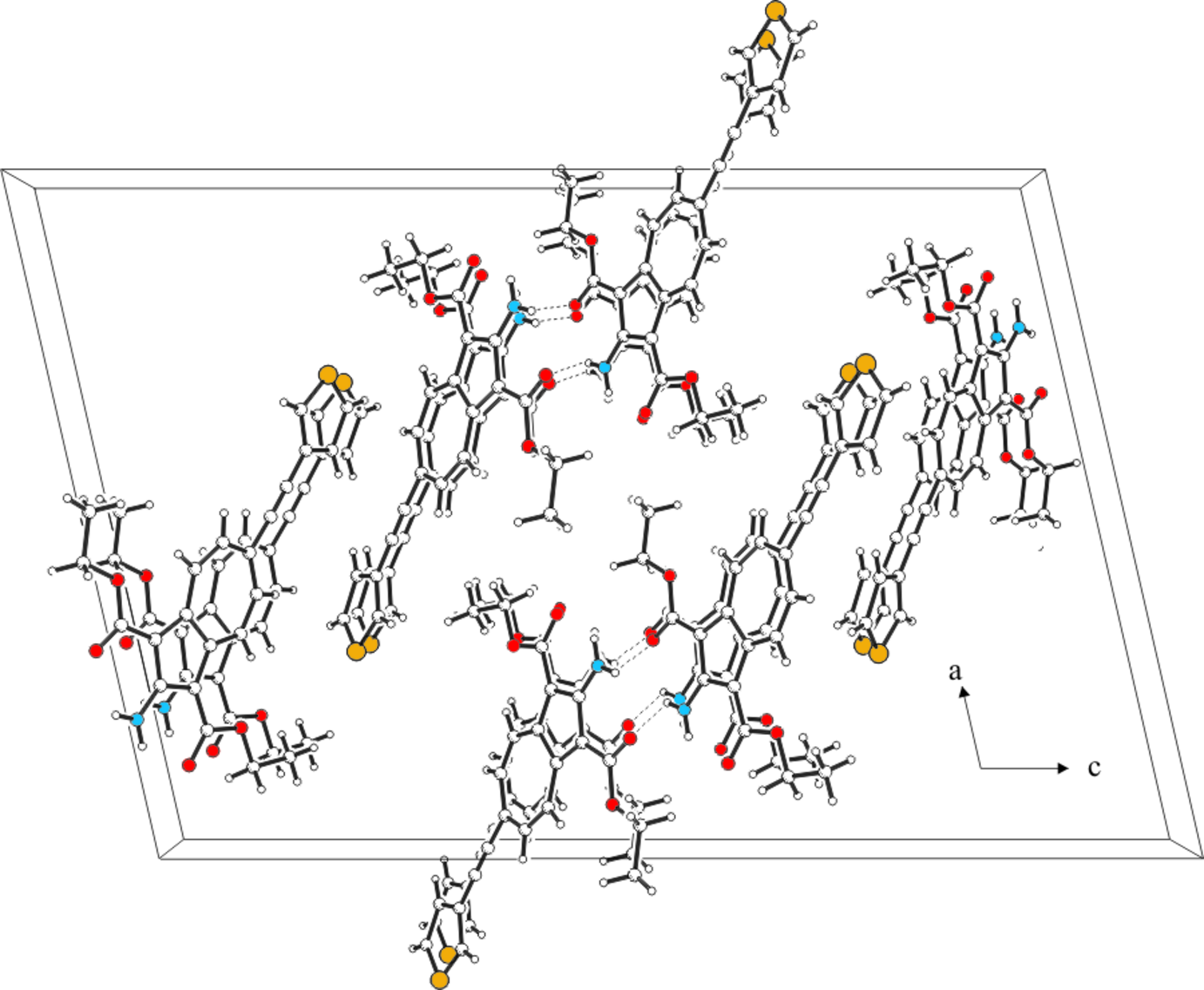

Supplement: Supplementary file 5 [file e-71-0o212-fig2.tif]
